# Anodal tDCS applied during multitasking training leads to transferable performance gains

**DOI:** 10.1038/s41598-017-13075-y

**Published:** 2017-10-11

**Authors:** Hannah L. Filmer, Maxwell Lyons, Jason B. Mattingley, Paul E. Dux

**Affiliations:** 1 School of Psychology, The University of Queensland, 4072 St Lucia, Australia; 2 Queensland Brain Institute, The University of Queensland, 4072 St Lucia, Australia

## Abstract

Cognitive training can lead to performance improvements that are specific to the tasks trained. Recent research has suggested that transcranial direct current stimulation (tDCS) applied during training of a simple response-selection paradigm can broaden performance benefits to an untrained task. Here we assessed the impact of combined tDCS and training on multitasking, stimulus-response mapping specificity, response-inhibition, and spatial attention performance in a cohort of healthy adults. Participants trained over four days with concurrent tDCS – anodal, cathodal, or sham – applied to the left prefrontal cortex. Immediately prior to, 1 day after, and 2 weeks after training, performance was assessed on the trained multitasking paradigm, an untrained multitasking paradigm, a go/no-go inhibition task, and a visual search task. Training combined with anodal tDCS, compared with training plus cathodal or sham stimulation, enhanced performance for the untrained multitasking paradigm and visual search tasks. By contrast, there were no training benefits for the go/no-go task. Our findings demonstrate that anodal tDCS combined with multitasking training can extend to untrained multitasking paradigms as well as spatial attention, but with no extension to the domain of response inhibition.

## Introduction

When participants practice a simple stimulus-response task, performance accuracy and speed typically improve. The benefits from such training appear to be very specific to the task(s) on which people train^1–4^. In the case of response-selection training, a recent study showed that training benefits do not even transfer to an identical task with different stimulus-response mappings^4^.

In recent years, there has been substantial interest in the potential for brain stimulation techniques, such as transcranial direct current stimulation (tDCS; see^5^), to facilitate cognitive performance^6–8^. Moreover, there is evidence that tDCS applied during training can enhance performance improvements for motor functions^9,10^, number processing^11,12^, working memory^13–15^ and decision-making^16^. To date, only a small number of tasks have been paired with tDCS during training. Thus, the range of processes that would potentially benefit from behavioural training and concurrent tDCS is unknown. In particular, stimulation has not been applied during the training of cognitive control, which is required for a wide range of tasks and, particularly, when multiple tasks must be performed concurrently, i.e. multitasking. Since multitasking is a key area of impairment in normal ageing^17^, and in a range of psychiatric and neurological conditions^18–20^, being able to maximise benefits from training could have important clinical applications.

Costs associated with performing multiple tasks concurrently, relative to single tasks, are thought to reflect the capacity limits of response-selection/decision-making^21^. We recently showed that tDCS applied to the left prefrontal cortex (PFC) during single-task decision-making training not only enhanced performance for the trained task, but also improved spatial attention performance as measured using visual search^16^. This transferred training benefit was not found when stimulation was applied alone (i.e., with no concurrent behavioural task), and was specific to left PFC stimulation (right PFC stimulation did not lead to training benefits). It remains unclear, however, whether such benefits from training with concurrent tDCS are observed for multitasking (although tDCS has been shown to modulate mutltiasking in single sessions^22–25^). In addition, in our previous study^16^, tDCS and training transfer were only assessed in a single untrained paradigm. Thus, the boundary conditions for the observed transfer have not been established, and it is unclear to what extent training combined with tDCS transfers across stimulus-response mappings and distinct cognitive domains.

To address these issues, we trained participants in a multitasking paradigm as they underwent concurrent tDCS. Participants received anodal, cathodal, or sham tDCS to the left PFC. Immediately prior to training, 1 day after training, and 2 weeks after training, participants completed a battery of four tasks. The untrained tasks were selected to measure the potential for “near” and “far” transfer^26^. For near transfer, a multitasking paradigm identical to that which participants trained upon was used, but with different stimuli. Previous research has shown that multitasking training alone does not lead to transfer to the same task with new stimuli^4^. For far transfer, visual search and go/no-go tasks were employed. These tasks tap distinct cognitive processes to multitasking (visual search – spatial attention; go/no-go – response inhibition), but visual search has been linked to activity in left prefrontal cortex^27^, while go/no-go is more commonly associated with activity in right PFC^28,29^. Hence, the two tasks vary in the extent to which the neural substrates overlap with the trained multitasking paradigm. By assessing whether the effects of combined training and tDCS generalise to one or both tasks, we were able to examine the extent and condition of training transfer following combined tDCS and cognitive control training.

## Results

To examine the key effects of stimulation and training on reaction times the data for each of the four tasks were submitted to ANOVAs with the between-subjects factor of stimulation group, and within-subjects factors including session (e.g. pre- and post-training). In addition, the baseline performance (pre-training) across the anodal, cathodal, and sham stimulation groups was subjected to a Bayesian ANOVA to provide stringent evidence for the null hypothesis (no difference before training between the stimulation groups).

### Baseline performance

**Figure 1:**
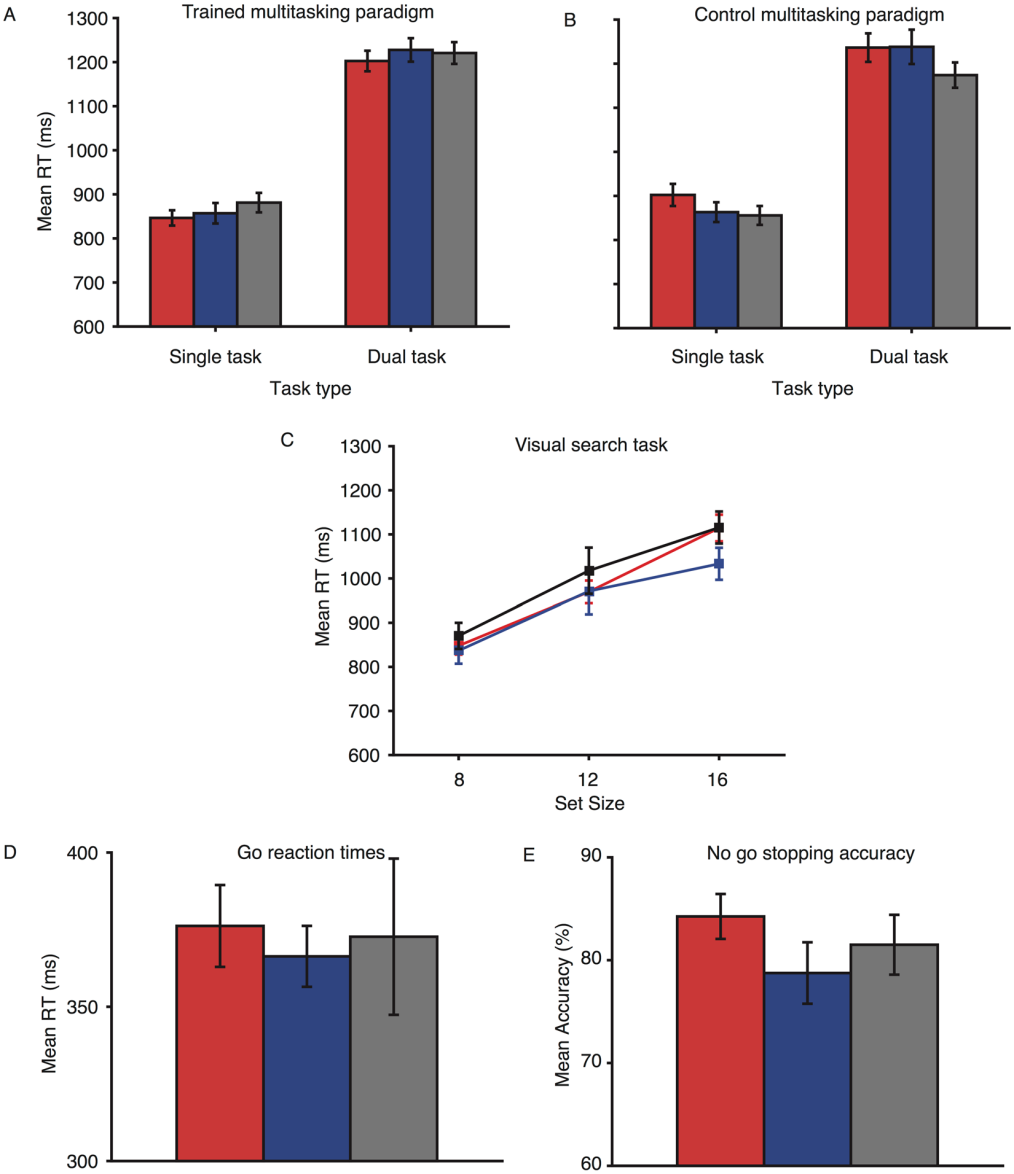
Baseline performance for the four tasks for each of the three stimulation groups. Red bars and lines represent the anodal group, blue the cathodal group, and black/grey the sham group. (**A**) Pre-training mean reaction times for the trained multitasking paradigm, shown for the single- and dual-task trials. (**B**) Pre-training mean reaction times for the transfer multitasking paradigm, shown for the single- and dual-task trials. (**C**) Pre-training mean reaction times for the untrained visual search task, per distractor set size. (**D**) Pre-training mean reaction times for the go responses on the go/no-go task. (**E**) Pre-training stop accuracy for the go/no-go task.

Overall, pre-training (baseline) performance was comparable for the three stimulation groups (see Fig. 1, and Tables 1 and 2). Specifically, the anodal, cathodal, and sham groups did not differ significantly in their pre-training reaction times for the trained multitasking paradigm (F(2, 56) = 0.86, p = 0.43, η^2^_p_ = 0.03, BF_10_ = 0.073), the untrained multitasking paradigm (F(2, 56) = 0.403, p = 0.67, η^2^_p_ = 0.014, BF_10_ = 0.139), the visual search task (F(2, 56) = 0.825, p = 0.444, η^2^_p_ = 0.029, BF_10_ = 0.206), or go-trial reaction times (F(2, 56) = 0.366, p = 0.695, η^2^_p_ = 0.013, BF_10_ = 0.185). The three groups also did not differ in their pre-training accuracy for the trained multitasking paradigm (F(2, 56) = 1.4, p = 0.26, η^2^_p_ = 0.05, BF_10_ = 0.424), the untrained multitasking paradigm (F(2, 56) = 0.67, p = 0.52, η^2^_p_ = 0.023, BF_10_ = 0.186), the visual search task (F(2, 56) = 0.45, p = 0.64, η^2^_p_ = 0.016, BF_10_ = 0.386), or no-go errors (F(2,56) = 1.04, p = 0.36, η^2^_p_ = 0.04, BF_10_ = 0.292). Go-trial error rates were not analysed as performance was at ceiling. These results confirm statistical equivalence at baseline testing for all the tasks. Thus, if we observe effects of combined training and stimulation, these cannot be attributed differences in pretraining performance across the groups, or to task exposure in the first session.

**Table 1:** Mean accuracy (%) for each session, for each of the four tasks. For the go/no-go task, accuracy is presented for the ‘go’ trials only (see text for no-go performance).

Task	Group	Pre	Training				Post	Follow up
			1	2	3	4		
Trained single/dual	Anode	93/87	93/89	92/91	93/91	93/92	93/92	92/91
	Cathode	90/84	91/86	92/88	92/87	92/91	88/85	*85/82*
	Sham	89/82	90/87	92/89	91/88	92/88	89/86	*83/86*
Control single/dual	Anode	91/84	—	—	—	—	90/88	92/90
	Cathode	92/84	—	—	—	—	87/83	*90/86*
	Sham	89/82	—	—	—	—	88/85	*85/81*
Visual search	Anode	96/96/96	—	—	—	—	97/97/96	96/97/96
	Cathode	95/96/94	—	—	—	—	95/96/95	*95/95/96*
	Sham	95/95/96	—	—	—	—	95/96/95	*94/96/96*
Go/no go	Anode	100	—	—	—	—	99.91	99.83
	Cathode	99.83	—	—	—	—	98.5	*99.92*
	Sham	99.82	—	—	—	—	100	*97.87*

The follow-up sessions comprised a slightly reduced N for the cathodal and sham groups (highlighted in italics).

**Table 2:** Mean reaction times (ms) for each session, for each of the four tasks and relevant conditions. For the go/no-go task, reaction times are presented for the ‘go’ trials only.

Task	Group	Pre	Training				Post	Follow up
			1	2	3	4		
Trained single/dual	Anode	846/1203	859/1184	776/1098	765/1082	770/1097	758/1068	824/1148
	Cathode	857/1228	861/1209	808/1148	804/1130	775/1113	791/1116	*884/1163*
	Sham	881/1221	858/1183	797/1110	798/1092	815/1113	813/1102	*864/1174*
Control single/dual	Anode	902/1237	—	—	—	—	792/1092	834/1183
	Cathode	863/1238	—	—	—	—	823/1141	*821/1139*
	Sham	856/1174	—	—	—	—	826/1097	*863/1139*
Visual search	Anode	848/970/1114	—	—	-	—	745/845/948	719/813/898
	Cathode	837/971/1033	—	—	—	—	743/872/955	*702/802/897*
	Sham	870/1018/1116	—	—	—	—	782/895/1013	*764/867/959*
Go/no go	Anode	376	—	—	—	—	363	363
	Cathode	366	—	—	—	—	356	351
	Sham	376	—	—	—	—	348	345

The follow-up sessions comprised a slightly reduced N for the cathodal and sham groups (highlighted in italics).

### Accuracy

Performance accuracy was analysed for all tasks, with the exception of go responses in the go/no-go task, as for these responses performance was at ceiling. For each of the tasks, stimulation group did not interact with any other factors (F < 2.35, p > 0.1 for all), with one exception: for the visual search task, from pre- to post-training, anodal tDCS improved accuracy for set size 8 only, whereas cathodal stimulation led to an improvement for set size 16 only (F(4,112) = 2.46, p = 0.049, η^2^_p_ = 0.08). These effects were not present when comparing pre-training performance to the follow-up session (F(4,108) = 1.83, p = 0.129, η^2^_p_ = 0.06). All analysis that follow were conducted on reaction times.

### Trained on multitasking paradigm

**Figure 2:**
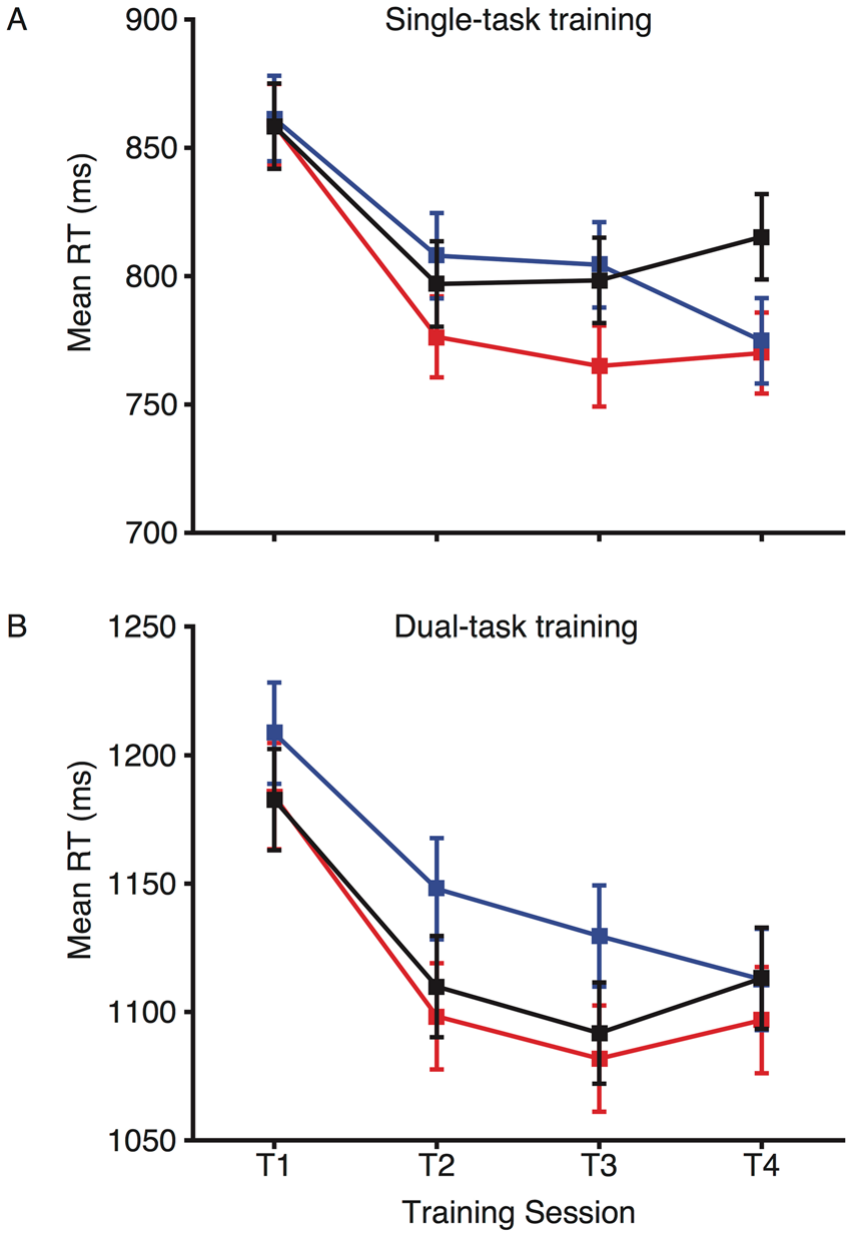
Mean reaction times across training sessions for the trained multitasking paradigm. Single task (**A**) and dual task (**B**) data are shown for the anodal (red), cathodal (blue), and sham (black) groups. Error bars represent the SEM for the change in reaction time from training sessions 1 to 4.

As predicted from previous work^16,30,31^, across the four training sessions there were substantial reductions in reaction times for both single- and dual-task trials (see Fig. 2; main effect of training session: F(3,168) = 23.359, p < 0.001, η^2^_p_ = 0.294). However, there were no differences in performance across the three stimulation groups (session × group interaction: F(6, 168) = 1.371, p = 0.229, η^2^_p_ = 0.047), and no stimulation effect that varied between the single- and dual-task trials (session × group × trial type interaction: F(6, 168) = 0.403, p = 0.876, η^2^_p_ = 0.014).

**Figure 3:**
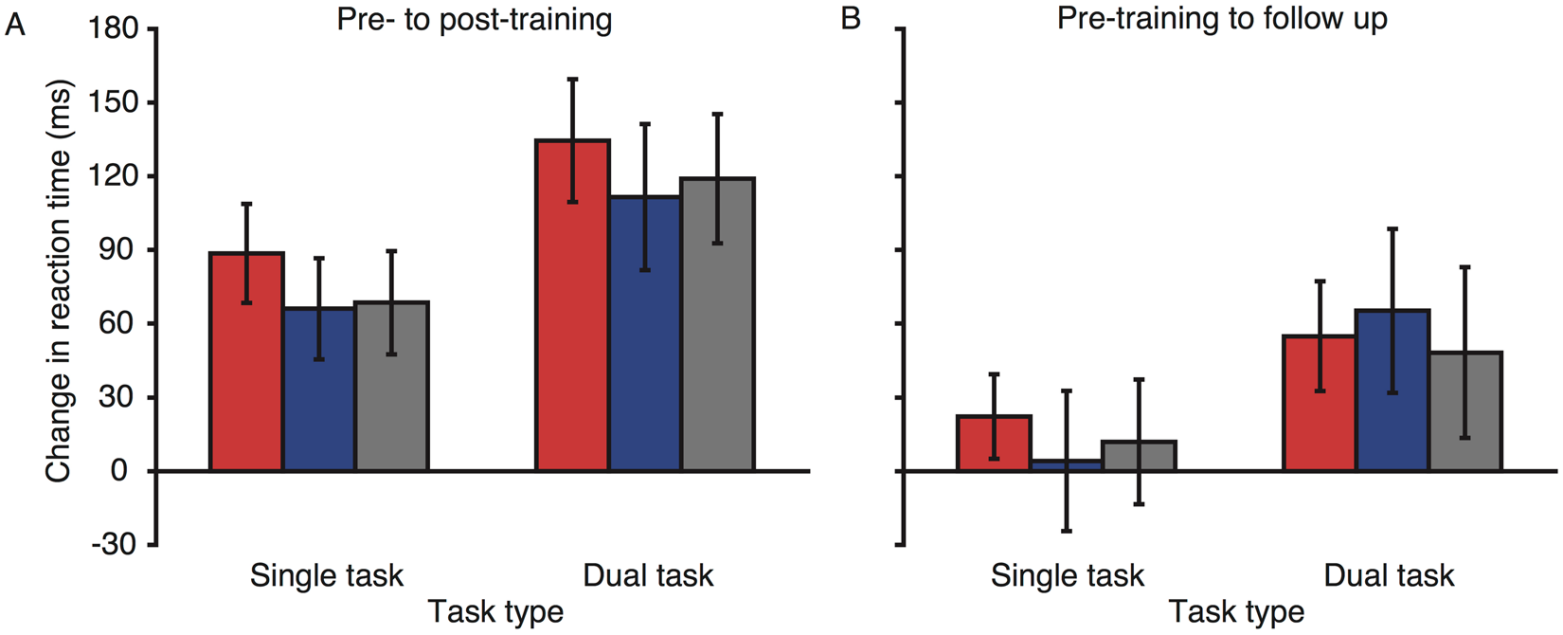
Change in reaction times from pre- to post-training (**A**), and pre-training to the follow up session (**B**), for the trained multitasking paradigm. Red bars represent the anodal group, blue bars the cathodal group, and grey bars the sham group. Error bars represent SEM for the change in reaction time.

Comparing the data from pre- to post-training, there was a clear multitasking cost, with longer reaction times for the dual- than single-task trials (mean difference = 333 ms; main effect of trial type: F(1, 56) = 859.004, p < 0.001, η^2^_p_ = 0.939). In addition, for all the stimulation groups, there was a marked improvement in reaction times from pre- to post-training (Fig. 3A, mean improvement = 98 ms, main effect of session: F(1, 56) = 59.185, p < 0.001, η^2^_p_ = 0.514). The dual-task trials showed greater benefit from training (122 ms) than single-task trials (73 ms; session × trial type interaction: F(1, 56) = 16.479, p < 0.001, η^2^_p_ = 0.227). Again, performance improvement with training was comparable for the three stimulation groups (session × group interaction: F(2, 56) = 0.297, p = 0.744, η^2^_p_ = 0.011), and this was true for both the single- and dual-task trials (session × group × trial type interaction: F(2, 56) = 0.018, p = 0.982, η^2^_p_ = 0.001).

To assess for any longer term benefits of training and stimulation, data from the pre-training and follow-up sessions were compared (see Fig. 3B). Fifty-six of the original participants completed the follow-up session two weeks after the final training session (anodal group: 20, cathodal group: 18, sham group: 18). Of those participants, one had a missing data set for the trained task. Of import, even after 2 weeks without training, the benefits to performance from training were still apparent, with all three groups showing a reduction in reaction times for the follow-up session (main effect of session: F(1, 52) = 5.939, p = 0.018, η^2^_p_ = 0.103), and this improvement was again greater for the dual-task trials (56 ms) than the single-task trials (17 ms; session × trial type interaction: F(1, 52) = 9.418, p = 0.003, η^2^_p_ = 0.154). There did not appear to be any added benefit from the application of tDCS, with comparable performance improvements for the three groups (all effects of stimulation: p > 0.3, η^2^_p_ < 0.05).

### Transfer multitasking paradigm

**Figure 4:**
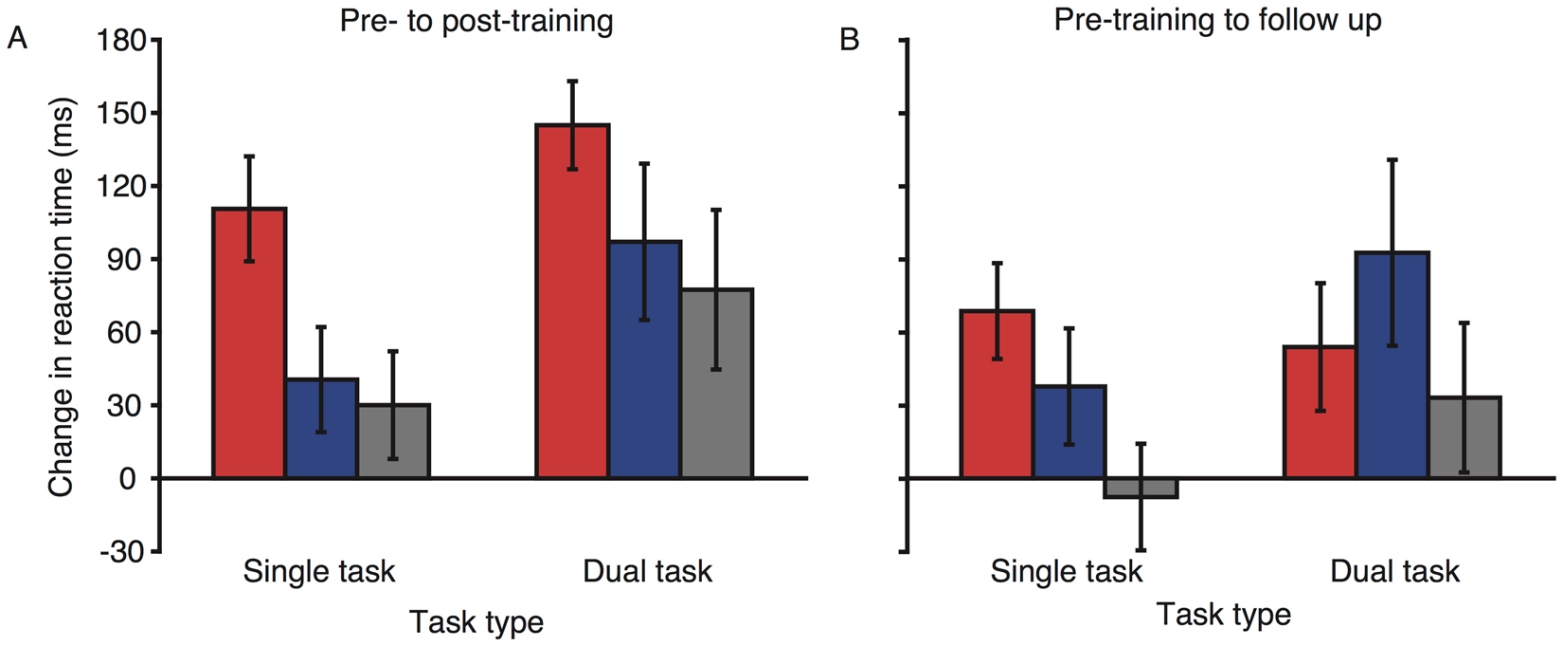
Change in reaction times from pre- to post-training (**A**), and pre-training to the follow up session (**B**), for the transfer multitasking paradigm. Red bars represent the anodal group, blue bars the cathodal group, and grey bars the sham group. Error bars represent SEM for the change in reaction time.

To ascertain whether stimulation induced near transfer of training benefits, performance for the untrained multitasking paradigm was compared from pre- to post-training (see Fig. 4A). Again, for this paradigm, overall responses were slower for the dual- than single-task trials (mean difference = 320 ms, main effect of trial type: F(1, 56) = 644.737, p < 0.001, η^2^_p_ = 0.92). All three groups showed improvement in reaction times across the sessions (mean improvement = 83 ms, main effect of session: F(1, 56) = 45.964, p < 0.001, η^2^_p_ = 0.451) with dual-task trials showing a greater improvement (106 ms) than single-task trials (60 ms; session × trial type interaction: F(1, 56) = 8.729, p = 0.005, η^2^_p_ = 0.135). Of import, however, for this paradigm transfer was observed. Specifically, there were greater improvements for the anodal (128 ms) relative to both the cathodal (69 ms) and sham (54 ms) tDCS groups (session × stimulation group interaction: F(2, 56) = 3.377, p = 0.041, η^2^_p_ = 0.108). This added benefit of anodal tDCS was consistent for the two trial types (session × stimulation group × trial type interaction: F(2, 56) = 0.173, p = 0.849, η^2^_p_ = 0.006). Thus, stimulation and training enhancements were observed for both the single- and dual-tasks.

Performance was compared from pre-training to the follow-up session to determine whether the training and stimulation benefits endured (see Fig. 4B). Whilst all groups showed improvement in the speed of responses (46 ms; main effect of session: F(1, 53) = 10.775, p = 0.002, η^2^_p_ = 0.169), the added performance benefits for the anodal stimulation group had reduced and were no longer significant (session × stimulation group interaction: F(2, 53) = 1.397, p = 0.256, η^2^_p_ = 0.05) relative to cathodal and sham stimulation.

### Visual search task

**Figure 5:**
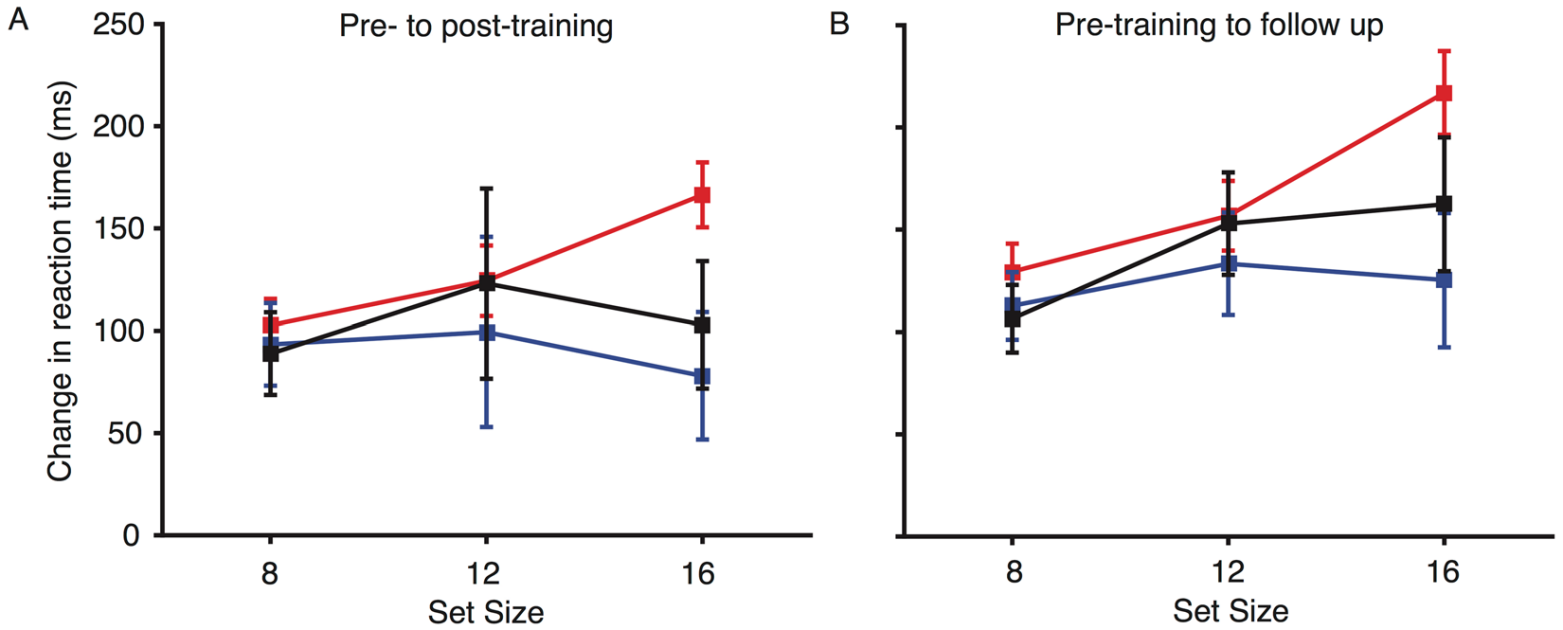
Change in reaction times from pre- to post-training (**A**), and pre-training to the follow up session (**B**), for the visual search task. Red lines represent the anodal group, blue lines the cathodal group, and black lines the sham group. Error bars represent SEM for the change in reaction time.

To address the possibility of far transfer, performance for the visual search task was compared from pre- to post-training (see Fig. 5A). Overall, there were substantial improvements in performance for all stimulation groups (mean improvement = 109 ms, main effect of session: F(1, 56) = 76.901, p < 0.001, η^2^_p_ = 0.579). As expected, reaction times increased with distractor set size (mean difference between set size 8 and 16 = 469 ms, main effect of set size: F(2, 112) = 364.25, p < 0.001, η^2^_p_ = 0.867). Given our previous results^16^, we predicted that anodal tDCS would lead to greater performance improvement, compared with sham and cathodal stimulation, for the larger set sizes. An ANOVA was conducted to compare visual search performance from pre- to post-training for the largest set size only (16). There was greater improvement in performance for the anodal (166 ms) relative to the sham (103 ms) and cathodal (78 ms) groups for set size 16 (session × stimulation group interaction: F(2, 56) = 3.2, p = 0.048, η^2^_p_ = 0.1). This finding replicates our previous work, but with a different trained task than that used previously.

Looking at the change in performance from pre-training to the follow-up session (Fig. 5B), all groups showed improvement in speed of responses (main effect of session: F(1, 53) = 100.796, p < 0.001, η^2^_p_ = 0.655), and this improvement was greater for the larger set sizes (session × set size interaction: F(2,106) = 11.078, p < 0.001, η^2^_p_ = 0.173). Stimulation group again modulated performance (session × stimulation group × set size interaction: F(4, 106) = 2.598, p = 0.04, η^2^_p_ = 0.089), with greater performance improvement at set size 16 for the anodal (217 ms) than the cathodal (125 ms) and sham (162 ms) groups. Hence, the performance enhancement for the anodal tDCS group at the largest set size, in our far transfer measure, remained present two weeks after cessation of training.

### Go/no-go task

**Figure 6:**
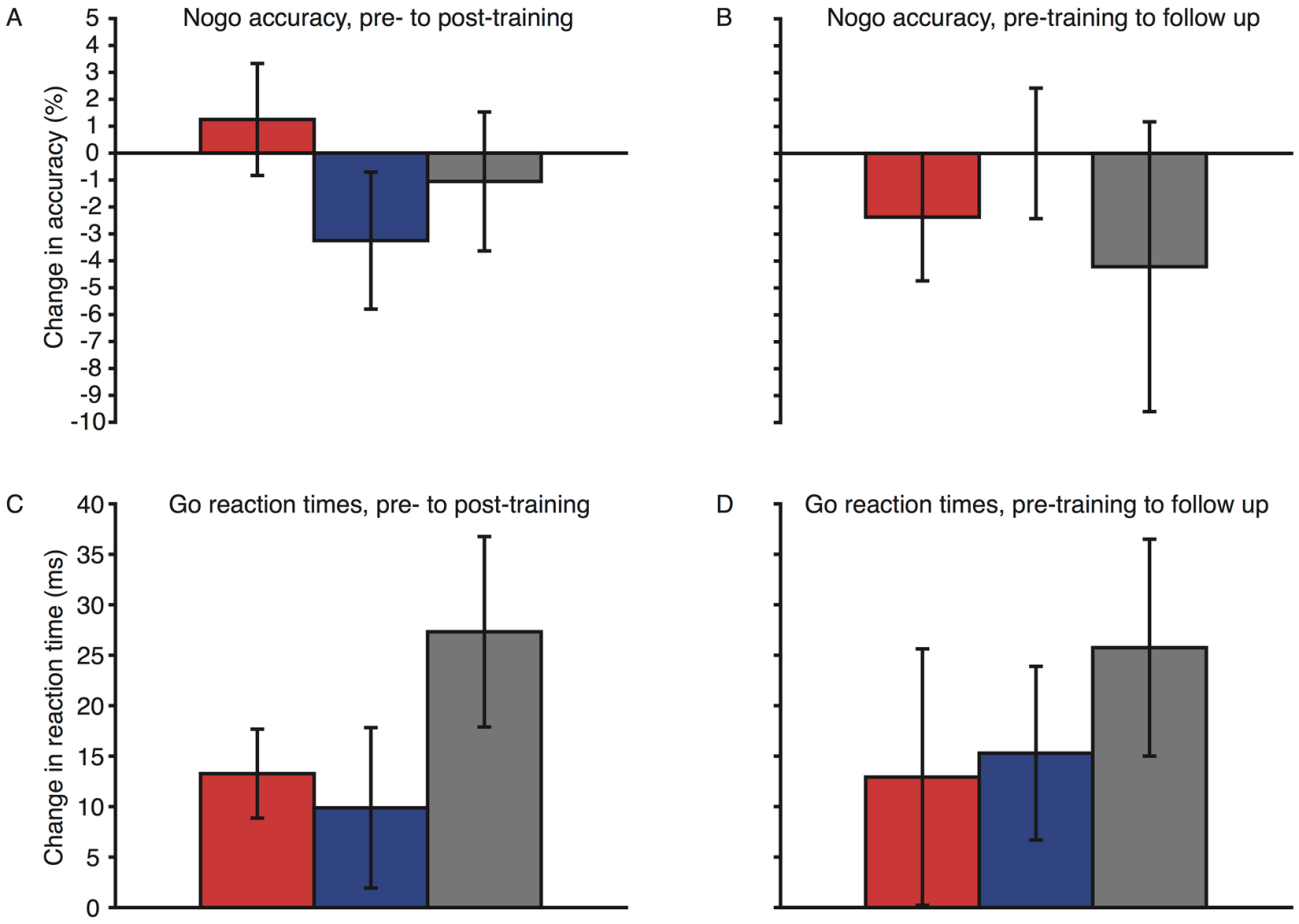
Change in reaction times from pre- to post-training (**A**), and pre-training to the follow up session (**B**), and change in no-go accuracy from pre-post training (**C**), and pre-training to the follow up session (**D**) for the go/no-go task. Red bars represent the anodal group, blue bars the cathodal group, and grey bars the sham group. Error bars represent SEM for the change in reaction time (**A** and **B**) or accuracy (**C** and **D**).

There were improvements in the reaction times for all groups (Fig. 6A, mean improvement = 17 ms, main effect of session: F(1, 56) = 15.113, p < 0.001, η^2^_p_ = 0.213). However, stimulation did not modulate response times (session × stimulation group interaction: F(2, 56) = 1.499, p = 0.232, η^2^_p_ = 0.05). Comparing reaction time performance from pre-training to the follow-up session (Fig. 6B), there remained an overall improvement (main effect of session: F(1, 52) = 7.578, p = 0.008, η^2^_p_ = 0.127), but, again there was no effect of stimulation group (session × stimulation group interaction: F(4, 52) = 0.355, p = 0.703, η^2^_p_ = 0.013). Hence stimulation did not modulate the go reaction times.

## Discussion

Our findings reveal transferable performance gains when anodal tDCS is applied to the left PFC during multitasking training. Anodal tDCS enhanced performance for two of the three transfer tasks: the multitasking paradigm that utilised untrained stimulus-response mappings, and a visual search task. Transfer effects were still evident at the two-week follow up for the visual search task. By contrast, for the go/no-go task, which involves inhibitory control and relies on activity within brain regions outside of those typically associated with multitasking^28,29^, there was no significant benefit from training and anodal tDCS.

It is possible that the performance gains shown by participants were not due to combined tDCS and training, but to tDCS alone. We believe this is unlikely, however, as a recent study conducted by our group using an analogous approach revealed no evidence for performance gains after multiple sessions of tDCS delivered over left PFC without a concurrent training task^16^. Given the relationship between decision-making and multitasking it seems unlikely tDCS alone would lead to the current pattern of results. In addition, our results are unlikely to be attributable to a general arousal effect, as the benefits we observed were specific to the application of anodal tDCS; cathodal stimulation did not lead to any significant training effects. Moreover, the fact that performance was not enhanced for the go/no-go task indicates that the influence of tDCS was not sufficiently general to improve all tested tasks (e.g., general modulations to sustained attention, or motivation). It is also unlikely the results reflect an effect of tDCS on low level perceptual or motor processes, as all tasks included visual or auditory sensory inputs and manual motor outputs, but not all were affected by combined training and stimulation. In addition, we have previously found modulations to training with tDCS to be unrelated to stimulus modality^22,31^. Finally, through response modelling, we have linked the modulations from combined training and tDCS to central task processes (evidence accumulation/drift rates, Filmer, *et al*.^16^).

We chose the left PFC as the target region because this area has previously been implicated in cognitive control, multitasking, and training^16,22,31,32^. Our study provides the first causal evidence for this region’s role in multitasking training, and converges with our earlier finding that left PFC plays a causal role in single-task decision-making training^16,31^. Of course, it must be considered that in the present study we targeted only one region of the cortex. Hence it is possible training benefits might be found with stimulation of other regions, and this should be the focus of future investigations. In previous work, we found that left- but not right-PFC stimulation yielded training and transfer benefits following combined single-task decision making training and anodal tDCS^16^. When taken together with the relevant neuroimaging literature, which suggests a key role for the left PFC specifically in cognitive control and multitasking^30,33–35^, it seems most likely that combined training and brain stimulation effects will be specific to the left PFC, but this remains to be determined.

We did not find any significant modulation of performance for the trained task with anodal tDCS. This is a different pattern of results to an earlier study conducted by our group, in which we observed trained-on task benefits from anodal stimulation^16^. It is possible that any benefits of stimulation for the trained task were masked by the training benefits present without tDCS, and consequently enhancement was only detected for the transfer tasks. Moreover, whereas Filmer *et al*.^16^ employed a relatively difficult 6-response alternative training task, here we used an easier 3-response alternative task, which might have reduced the potential for further improvement above and beyond training alone. Finally, it is also possible that the current study was somewhat underpowered. Although we determined the sample size a priori, based on our own recent findings from a paired tDCS and training study^16^, it is possible that the training effect in the present study was smaller and simply required a larger sample to be detected. Regardless, we can conclude with a high degree of certainty that one of the critical effects of anodal tDCS over left PFC is to generalise the training benefit to a broader, but not universal, set of tasks/processes. That is to say, while the present results are statitistically significant, but modest in terms of effect size, they converge with the observations of Filmer *et al*.^16^ in demonstrating sustained transfer of practice following left PFC tDCS.

Typically, when one set of stimulus-response mappings is trained upon, there is no transfer of the training benefit to a multitasking paradigm with different stimuli^4,36^. In the current study, the application of anodal tDCS generalised the benefits of training, suggesting that learning under these conditions is not bound to specific stimulus-response mappings. Furthermore, the transferred training gains to visual search, conceptually replicating our earlier finding^16^, supports the broad transfer of learning via tDCS. Indeed, visual search has been linked to activity in the left PFC^27^, and yet the other far transfer task, the go/no-go paradigm, which did not show any transfer benefits, is more commonly associated with the right PFC^28,29^. A potential explanation for our findings, therefore, is that tDCS modulates activity within the left PFC which in turn facilitates performance for tasks that tap the same prefrontal cortical area. Alternatively, there might be a single mechanism for the performance enhancement that encompasses dual- and single-task processes, as well as visual search, but not response inhibition. This mechanism could represent a task-general process such as adaptive tuning of neurons to evidence accumulators for the relevant context or task^37^.

The transferable benefit of anodal tDCS remained present 2 weeks after cessation of training for the visual search task, but was less persistent for the control multitasking paradigm. Thus the longevity of the transferable benefits reported here was variable. The current findings are broadly in line with our previous study^16^, in which a response selection task showed a weakened (non- significant) benefit at 2-week follow-up, but a visual search task showed sustained (and significant) performance enhancement. It is possible that increasing the number of training sessions, or modifying the tDCS parameters (e.g., increasing stimulation intensity) might lead to longer lasting effects for near transfer tasks. It is also possible that visual search provides a more sensitive measure of the processes modified by combined anodal tDCS and training.

In conclusion, we here mapped the extent of improvement in an aspect of cognitive control via tDCS applied during training. We show that left prefrontal stimulation combined with concurrent training can improve performance for cognitive control operations associated with multitasking, and this improvement generalises to both near and far cognitive operations. Importantly, performance gains were generalised, but not universal – there was no improvement in response inhibition – illustrating important boundary conditions to the effects of combined training and brain stimulation. Transfer effects were specific to anodal tDCS, which may influence activity within the left PFC. Collectively, this study demonstrates the potential for tDCS as a tool for enhancing training outcomes in individuals with impaired cognition, and specifically operations associated with cognitive control that are particularly susceptible to the effects of disease and ageing.

## Materials and Methods

### Participants

Eighty-seven right-handed participants from The University of Queensland took part in the study. All participants had normal hearing, and normal or corrected to normal vision, and successfully passed safety screening, which included questions relating to medication use. Four participants were excluded due to missing data, and a further 24 were excluded for poor pre-training performance (>40% errors). Exclusion criteria were established a priori, and exclusions based on participants’ pre-training performance occurred without knowledge of the training outcome. The final sample consisted of 59 participants, split into three groups (20, 20 and 19 participants in each). Allocation to group was pseudo-random for most of the experiment, with participants being allocated to one of the three groups in turn. However, the last few participants were allocated to specific groups to ensure approximately equivalent group sizes following exclusions. The mean age of the final sample was 21 years (SD = 2 years; 51 women), and was comparable for the anodal (21 years, 18 women), cathodal (21 years, 18 women), and sham (21 years, 15 women) groups. The University of Queensland Human Research Ethics committee approved the study, all participants gave informed, written consent, and the study was run in accorandance with the Declaration of Helsinki.

### Stimulation protocol

**Figure 7:**
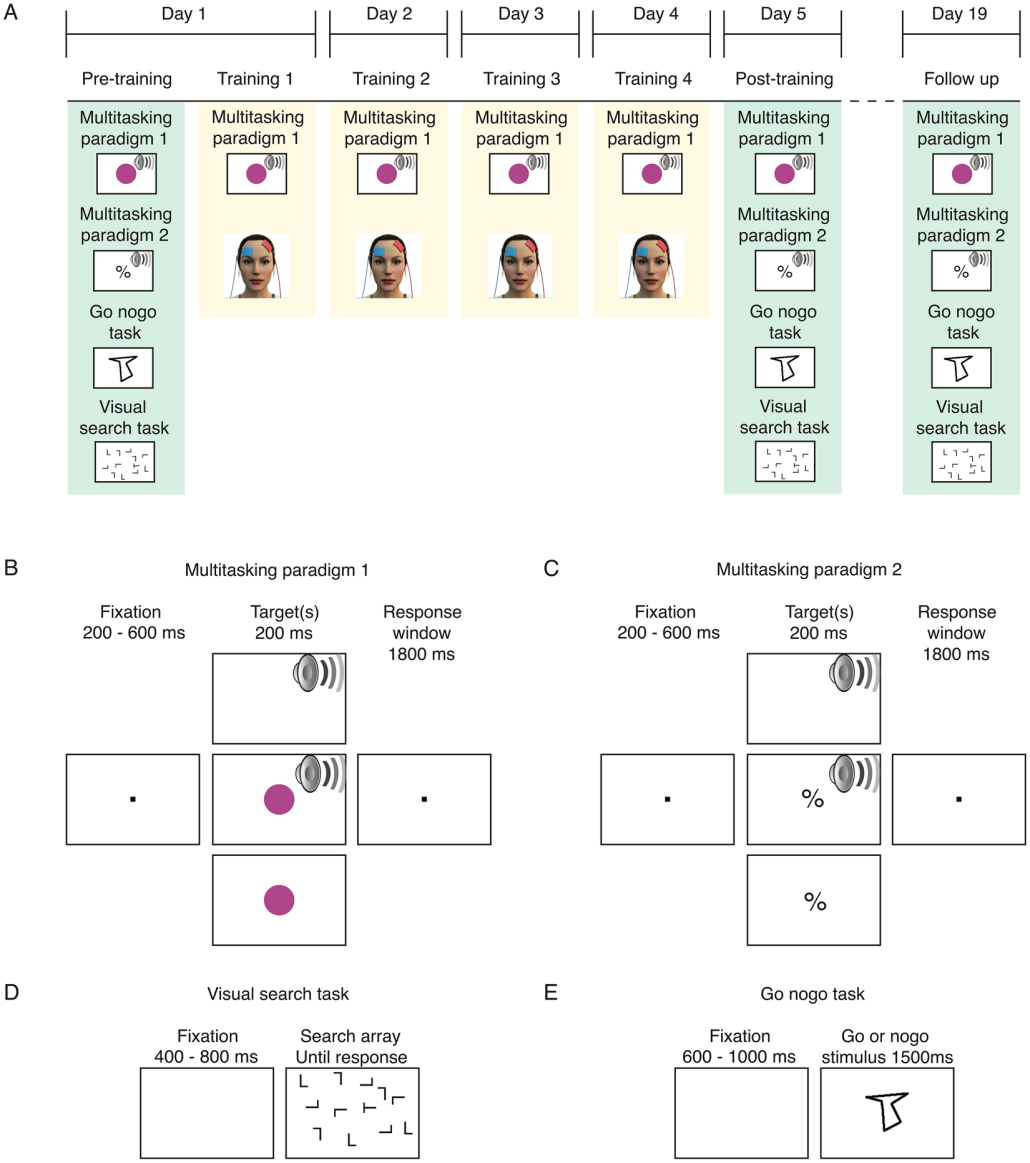
Experiment design overview. (**A**) Outline of the experimental sessions. Participants undertook a pre-training session in which they completed practice, and a full experimental block, for the trained and the transfer tasks. Participants then completed four sessions, on consecutive days, during which they undertook the trained task with concurrent tDCS. Two post-training sessions were then conducted one day after, and two weeks after, the cessation of training, in which participants again completed all four tasks. (**B**) Trial outline for the trained on multitasking paradigm. (**C**) Trial outline for the untrained (transfer) multitasking paradigm. (**D**) Trial outline for the untrained (transfer) visual search task. (**E**) Trial outline for the untrained (transfer) go/no-go task.

The stimulation protocol closely followed that of Filmer *et al*.^16^ who observed transferable gains following single-task decision-making training paired with anodal stimulation of left lateral PFC. Each participant completed four sessions on consecutive days, with tDCS (Neuroconn DC-Stimulator) delivered to the left PFC. The stimulation site was localised via the EEG 10–20 system^38^, with the target electrode placed 1 cm posterior to F3^16,22,31^. The reference electrode was placed over the contralateral orbitofrontal region (see Fig. 7A). Both electrodes were 5 × 5 cm saline soaked sponges. The stimulation intensity was 0.7 mA, resulting in a current density of 0.028 mA/cm^2^. We employed this stimulation intensity as we had previously found it was effective in promoting effects with single-task training^16^. The experiment followed a single blinded, between-group design, with each of the three groups of participants receiving a different type of stimulation – anodal, cathodal, or sham. The anodal and cathodal groups received stimulation for a total of 13 minutes, including 30 seconds of the current ramping up and down. The sham group received a total of 1 minute 15 seconds of stimulation, including a 30 second ramp up and down^16,22,31,39^.

### Trained multitasking paradigm

Participants trained on a sensory-motor paradigm consisting of three different trial types: single visual task, single auditory task, and dual-task (see Fig. 7B). For the single task trials, either a coloured circle (visual) or a sound (auditory) was presented for 200 ms, and participants had to respond as quickly and accurately as possible to the identity of the stimulus. Responses were made via a keyboard. There were three different possible colours (red: RGB 237 32 36, dark green: RGB 10 130 65, dark blue: RGB 44 71 151), and three possible complex tones^16,22,30,31,33^, each associated with a different key on the keyboard. For the dual-task trials, both a sound and a colour were presented simultaneously. Participants were instructed to respond to both stimuli as quickly and as accurately as possible. The three trial types were randomly intermixed within blocks of trials.

### Transfer tasks

The experiment also consisted of three tasks to assess transfer of training effects (see Fig. 7C-E). The first transfer task was an alternate version of the trained multitasking paradigm, but with different visual and auditory stimuli. The visual stimuli consisted of symbols (&, #, and %), and the auditory stimuli were three complex tones that were different from those used in the trained task^16,22,30,31,33^. This task tested the extent to which the training and stimulation parameters generalised to different sensory-motor mappings within the broad multitasking context. As noted above, when such training is performed alone, without concurrent brain stimulation, no transfer is observed when stimuli in the multuitasking paradigm are changed (e.g., Garner *et al*., 2014). The second transfer measure assessed spatial attention with a visual search task, where participants had to locate and respond to a letter ‘T’ amongst distractor ‘L’ stimuli. Once located, participants indicated whether the T was oriented through 90 or 270 degrees. The number of distractor ‘L’s was 8, 12, or 16, and these trial types were randomly intermixed. The third transfer task was a go/no-go paradigm assessing the cognitive control operation of inhibition, which is distinct from that tapped by multitasking^40^. For this task, an abstract line shape was shown on each trial (see Fig. 7E) and participants had to press the ‘g’ key on the keyboard if it was the ‘go’ stimulus, or withhold their response if it was the specific ‘no-go’ stimulus. The go stimulus was presented on 75% of trials. Participants were instructed to respond as quickly and as accurately as they could in all three tasks, with responses made via a keyboard.

### Procedure

An outline of the experimental sessions is shown in Fig. 7A. During the experiment, participants sat approximately 70 cm from a 19″ CRT monitor with a refresh rate of 100 Hz. They initially completed all four tasks – including practice and a baseline measurement for each (baseline measures: trained task = 240 trials, transfer multitasking paradigm 1 = 240 trials, visual search = 240 trials, go/no-go = 80). The trained task, transfer multitasking paradigm, and visual search took approximately 10 minutes to complete, and the go/no-go took approximately 5 minutes to complete. The order in which the tasks were completed was controlled across participants using a Latin square. The electrodes were then fitted and the first training session completed. For the first 10 seconds of stimulation, participants were asked to sit quietly and were reminded of the response mappings for the two tasks. For the following 10 seconds participants were shown the three response keys for the sound stimuli, and could hear the sounds if they pressed a relevant key to remind themselves of the stimulus-response mappings. After this, participants had another 10 seconds where the three response keys for the colour task were shown, with the relevant colour displayed underneath each key. The main task began at the same time as the current ramp-up ceased. The task then lasted for just over 12 minutes, and consisted of 240 trials (80 single-auditory, 80 single-visual, and 80 dual-task). The task was completed 10 seconds into the current ramp down, and participants sat quietly for the remaining 20 seconds of the ramp down. For each of the next three days, participants again undertook the trained task with concurrent stimulation. For the post-training session (Day 5) and the follow up session (2 weeks later), participants completed all four tasks again, but with no brain stimulation.
